# Laparoscopic Radical Prostatectomy Performed in a Patient With Zinner’s Syndrome: The First Case Described in the Literature

**DOI:** 10.7759/cureus.33764

**Published:** 2023-01-14

**Authors:** Theodoros Spinos, Ioannis Leotsakos, Ioannis Katafigiotis, Filippos Nikitakis, Markos Karavitakis

**Affiliations:** 1 Department of Pathology, National and Kapodistrian University of Athens, Athens, GRC; 2 Department of Laparoscopy and Endourology, Central Urology, Lefkos Stavros - The Athens Clinic, Athens, GRC

**Keywords:** surgical technique, radical prostatectomy, laparoscopic, prostate cancer, zinner’s syndrome

## Abstract

Zinner’s syndrome is a rare congenital disorder presenting with unilateral renal agenesis or dysgenesis, ipsilateral seminal vesicle cysts, and ejaculatory duct obstruction. Treatment of this syndrome can be conservative or surgical. In this case report, we describe the case of a 72-year-old patient who was diagnosed with Zinner’s syndrome and underwent laparoscopic radical prostatectomy for prostate cancer treatment. The peculiarity of our case was that the patient’s ureter emptied ectopically into the left seminal vesicle, which was notably enlarged and multicystic in appearance. Although many minimally invasive procedures have been reported for treating symptomatic Zinner’s syndrome, to our knowledge, this is the first reported case of prostate cancer in a patient with Zinner’s syndrome who was treated with laparoscopic radical prostatectomy. Laparoscopic radical prostatectomy can be safely and efficiently performed in patients with Zinner’s syndrome and synchronous prostate cancer by urological surgeons with extensive experience in laparoscopy in high-volume centers.

## Introduction

Zinner’s syndrome is a rare congenital disorder, which was first described by Zinner in 1914, consisting of a triad of mesonephric (Wolffian) duct abnormalities. This triad includes unilateral renal agenesis or dysgenesis, ipsilateral seminal vesicle cysts, and ejaculatory duct obstruction [1]. Although malformations are usually ipsilateral, contralateral variations have also been described. In most cases, this syndrome is asymptomatic and found incidentally during investigation for other reasons [2]. The pathogenesis of the syndrome can be traced back to embryogenesis between the fourth and thirteenth gestational week. Irregular development of the distal part of the Wolffian duct results in abnormal maturation of the kidney and the ejaculatory duct [3]. Symptoms of Zinner’s syndrome usually present during the third or fourth decade of life [1]. Van den Ouden et al., studying 52 patients diagnosed with Zinner’s syndrome, reported that dysuria (37%), frequency (33%), perineal pain (29%), epididymitis (27%), and pain after ejaculation (21%) were the most common symptoms [4]. Other symptoms include hypogastric pain, hemospermia, and infertility. Interestingly, large seminal vesicle cysts can be palpable during rectal examination [2].

Asymptomatic patients can be simply observed. In the case of mild symptoms, percutaneous drainage and transurethral or transrectal aspiration of the ureterocele or seminal vesicle cyst can be performed. However, only surgical removal of the cyst and the seminal vesicle is 100% efficient. When more severe symptoms are present, more invasive procedures should be employed. Open surgery, performed through a transvesical, retrovesical, transperineal, or transcoccygeal approach, was historically the first treatment modality that was associated with a high success rate. Nowadays, transurethral, laparoscopic, and robotic-assisted approaches are also performed to treat Zinner’s syndrome [5,6]. Abdominal ultrasound can provide information about the composition, dimensions, and exact location of a mass; the bladder’s integrity; and the presence of ipsilateral renal agenesis or dysgenesis. CT urogram can confirm the absence of the ipsilateral kidney and ureter, while cystoscopy can detect anatomical malformations of the bladder and urethra, including the absence of the ipsilateral hemitrigone and intravesical cyst protrusion. Nevertheless, MRI remains the gold standard diagnostic tool, providing detailed imaging of cyst contents [2,7]. In this case report, we describe the case of a 72-year-old patient who was diagnosed with Zinner’s syndrome and underwent laparoscopic radical prostatectomy (LRP) for prostate cancer treatment. There are currently around 200 reports of Zinner’s syndrome in the literature. Although several articles report the use of minimally invasive approaches, for treating abnormalities associated with Zinner’s syndrome, to our knowledge, this is the first reported case of prostate cancer in a patient with Zinner’s syndrome who was treated with LRP.

## Case presentation

**Figure 1:**
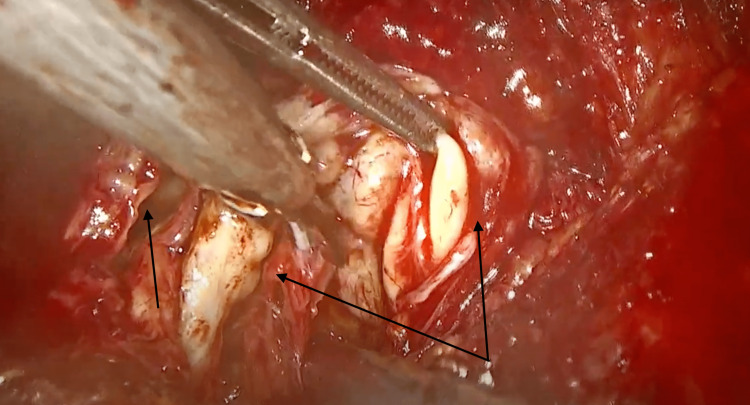
Left ureter, left ejaculatory duct, and seminal vesicle.

A 72-year-old man presented in our department with a high prostate-specific antigen (PSA) value (4.42 ng/mL), which was found incidentally during screening. The digital examination was negative. Multiparametric MRI imaging revealed a lesion on the left side of the prostate, with a Prostate Imaging Reporting and Data System (PI-RADS) score of 4. A transperineal fusion biopsy of the lesion confirmed the presence of prostatic adenocarcinoma with a Gleason score of 3 + 4. TNM staging was cT2N0M0. All radical prostatectomies in our center are performed laparoscopically using three-dimensional (3D) vision. The procedure was performed by a highly skilled urological surgeon, who has performed over 500 LRP procedures, in a high-volume center, while an anesthesiologist highly skilled in laparoscopic surgeries was employed. The peculiarity of our patient was that he was diagnosed with asymptomatic Zinner’s syndrome. Moreover, his left ureter emptied ectopically into the left seminal vesicle, which was notably enlarged and multicystic in appearance (Figure 1). Preoperative MRI revealed left seminal vesicle ectasia, left renal agenesis, and ectopic implantation of the left ureter into the left seminal vesicle.

**Figure 2:**
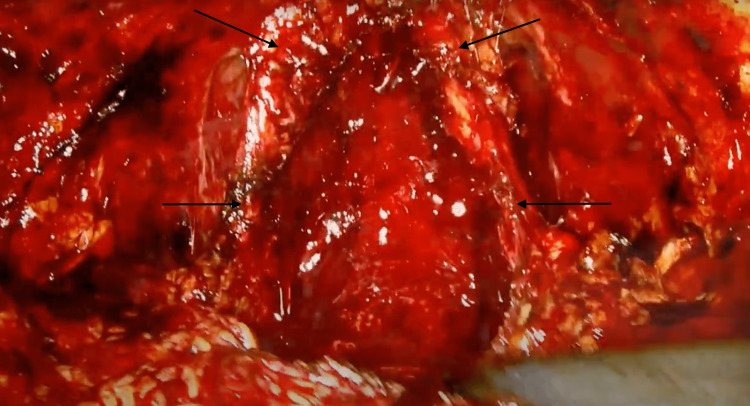
Puboprostatic ligament preservation and neurovascular bundles (intrafascial high-release preservation technique).

**Figure 3:**
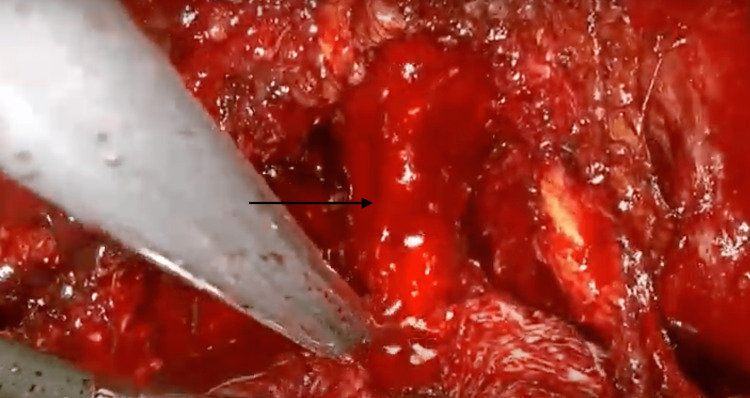
Maximum urethral length preservation technique.

**Figure 4:**
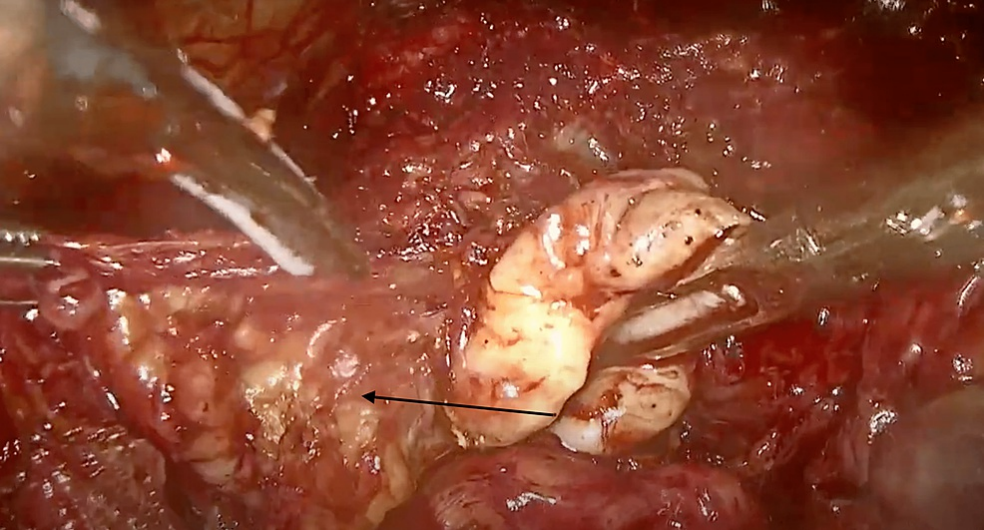
Left seminal vesicle ectasia.

**Figure 5:**
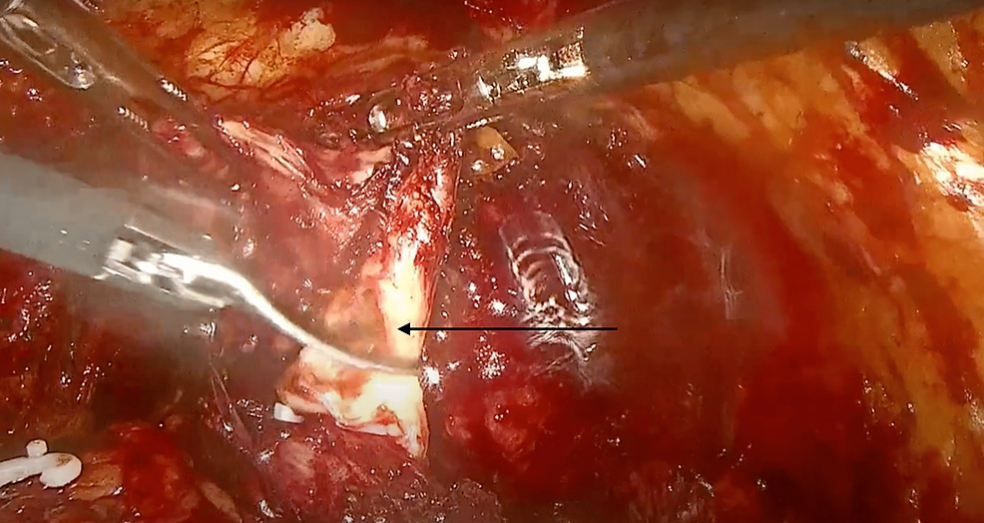
The ligated left ureter (ipsilateral renal agenesis).

Key points of our technique were: (1) placement of five trocars; (2) preparation of the extraperitoneal space and of the space of Retzius; (3) opening of the endopelvic fascia; (4) preparation of the bladder neck and its preservation using our signature technique (which taking advantage of the catheter traction fully preserved the bladder neck in a circumferential manner); (5) preparation and dissection of vas deferans and seminal vesicles; (6) total preservation of the neurovascular bundles (Figure 2) using the “veil of Aphrodite” technique (we used a clipless technique for nerve sparing, employing ultrasound scissors); (7) preparation of the apex of the prostate using the “hood” technique (Figure 2), and dissection of the urethra with maximal preservation of membranous urethral length using the “collar” technique (Figure 3); and (8) vesicourethral anastomosis with “V lock” 3/0 sutures. The operation was completed in 110 minutes. During the preparation of the left seminal vesicle, an area of ectasia was evidenced (Figure 4), in which the ipsilateral ureter ended up. We decided to ligate the left ureter, given that the patient had ipsilateral kidney agenesis (Figure 5).

**Figure 6:**
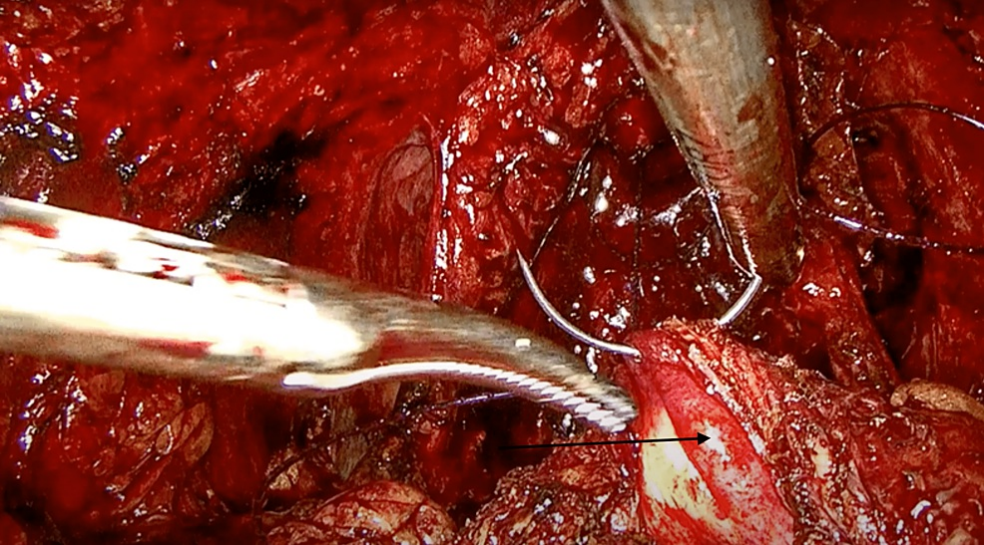
Malformations of Zinner’s syndrome leading to a positioning of the right ureteric orifice in very close proximity to the bladder neck.

Moreover, malformations of Zinner’s syndrome led to a positioning of the right ureteric orifice in very close proximity to the bladder neck (Figure 6).

The patient’s intraoperative and postoperative courses were uneventful and he was discharged from the hospital on the second postoperative day. The pathologist’s report confirmed the ectasia of the left seminal vesicle and the presence of a part of the ipsilateral ureter into it. The pathological TNM was pT3aNxMO. The patient was fully continent after catheter removal on postoperative day 10 and was using zero pads during the day.

## Discussion

Many different minimally invasive procedures for symptomatic Zinner’s syndrome have been reported. Historically, open vesiculectomy, using a transperineal or a transabdominal approach, was the definitive surgical treatment for symptomatic seminal vesicle cysts [1]. Kavoussi et al. demonstrated that the seminal vesicles can also be approached laparoscopically and showed the way how laparoscopy should be employed to treat retrovesical lesions [8]. Better visualization of pelvic anatomical structures using high magnification, shorter hospital stays, and faster patient recovery have rendered minimally invasive approaches into the surgical treatments of choice for seminal vesicle diseases [1,8].

Kord et al. operated on five patients who presented with symptomatic Zinner’s syndrome (a laparoscopic approach was used for four patients and a robotic-assisted one for one patient), reporting that a minimally invasive approach is not only feasible and effective but also very beneficial for both the patient and the surgeon [7]. Similarly, Maehana et al. presenting a laparoscopic approach for a case of Zinner’s syndrome concluded that the laparoscopic approach provides a satisfactory surgical field, avoiding symptom recurrence associated with the selected puncture of the ureterocele or the seminal vesicle cyst [5]. Ploumidis et al., describing the case of a 45-year-old male with a symptomatic 17.2 cm seminal vesicle cyst, which was removed using a robotic-assisted approach, outlined that 3D vision and wristed instrumentation of the robotic system enable safe excision of large seminal vesicle cysts [9]. Likewise, Demaeyer et al. decided to use a robotic-assisted approach to completely remove the cyst and the right seminal vesicle of a 33-year-old man with symptomatic Zinner’s syndrome. They suggested that minimally invasive approaches, similar to conventional and robotic-assisted laparoscopy, should be considered the gold standard surgical treatments for this condition, given their safety and effectiveness [6].

Many other remarkable minimally invasive approaches for the treatment of symptomatic Zinner’s syndrome have been reported [1,10-14]. In our case, laparoscopy was not used to treat the symptoms of Zinner’s syndrome or any medical situation associated with it but for prostate cancer. To our knowledge, this is the first case of an LRP procedure performed for a patient with Zinner’s syndrome described in the literature. As already mentioned, our patient had a remarkable variant of the syndrome, with his left ureter ending up directly in the ipsilateral seminal vesicle. High magnification, 3D vision, and precise surgical movements associated with 3D laparoscopy enabled accurate and safe recognition, preparation, and dissection of anatomical structures in the pelvis. We strongly believe that conventional open surgery would be very difficult and unsafe for treating this particular patient.

## Conclusions

In patients with Zinner’s syndrome and synchronous prostate cancer, LRP can be safely and efficiently performed by urological surgeons with extensive experience in laparoscopy in high-volume centers. High magnification, used during laparoscopy, facilitates precise recognition and meticulous dissection of anatomical structures in the pelvis when challenging anatomical variations are present.
